# Neonicotinoid contamination in wildflowers collected from citrus orchards in a northwestern Mediterranean Region (Spain) after tree foliar treatments

**DOI:** 10.1007/s11356-022-19331-7

**Published:** 2022-03-14

**Authors:** Ana Isabel García-Valcárcel, José Miguel Campos-Rivela, María Dolores Hernando Guil, María Teresa Martínez-Ferrer

**Affiliations:** 1 Department of Environment and Agronomy, National Institute for Agricultural and Food Research and Technology – INIA-CSIC, Ctra. La Coruña Km. 7.5, 28040 Madrid, Spain; 2 Institute of Agrifood Research and Technology, IRTA, 43870 Amposta, Tarragona, Spain

**Keywords:** Imidacloprid, Thiamethoxam, Wildflowers, Citrus-orchard, Foliar application, Mediterranean conditions

## Abstract

Ground-cover vegetation attracts and harbors beneficial insects to the agrosystem, playing an important role in conservation biological control. Integrated pest management (IPM) program guidelines recommend the implantation of sowed or resident wild covers in perennial crops. Given the high-quality fruit requirements, even in IPM programs, insecticides can be required in citrus crops. This study presents, over a year, the levels of neonicotinoids (thiamethoxam and imidacloprid) in not-target ground-cover wildflowers growing spontaneously in citrus orchards after foliar treatment of citrus trees. The presence and persistence of these neonicotinoids in different wildflower species were studied. Concentrations of thiamethoxam and imidacloprid in whole wildflowers ranged from < method quantification limit (MQL) to 52.9 ng g^−1^ and from < MQL to 98.6 ng g^−1^, respectively. Thiamethoxam was more frequently detected than imidacloprid. Thiamethoxam and imidacloprid were detected up to 336 and 230 days after treatment, respectively. The highest detection frequencies (100%) and highest thiamethoxam and imidacloprid mean concentrations (26.0 ± 7.3 ng g^−1^ and 11.0 ± 10.6 ng g^−1^, respectively) occurred in wildflowers collected 9 days after the treatments. Since application, a clear decrease in the concentration of both compounds and differences in the accumulation depending on wildflower species were observed. Cross contamination was detected, indicating a transport from adjacent treated plots. Maintaining a cover crop in citrus orchards may lead to detrimental effects on non-target arthropods if these neonicotinoid compounds are used for pest control since they can entail a chronic exposure during at least 230 days for imidacloprid and 336 days for thiamethoxam.

**Supplementary Information:**

The online version contains supplementary material available at 10.1007/s11356-022-19331-7.

## Introduction

The integrated pest management (IPM) system relies on a combination of biological, mechanical, and chemical strategies to keep pest populations below economic threshold (Barzman et al. 2015). Conservation biological control, which aims to protect and enhance the effectiveness of natural enemies by modifying the environment or existing practices (Eilenberg et al. 2001), is a key component of citrus IPM programs. In citrus agrosystems, most of the pest species are naturally controlled by associated entomofauna. Therefore, conservation biological control is crucial. *Hymenoptera*, *diptera*, *coleoptera*, phytoseiid mites, *coniopterigidae*, or *chrysopidae* (Garcia-Marí 2012; Jacas and Urbaneja 2010; Martínez-Ferrer et al. 2015) are important regulators of citrus pests and, in many cases, it is not necessary to perform chemical treatments respecting these important pest/natural enemies’ equilibria.

As an ecological infrastructure, ground cover plays an important role in conservation biological control and in IPM programs for perennial crops. Thus, the implantation of sowed or resident wild cover is recommended (IOBC/WPRS, 2016). Currently, most of the citrus orchards keep a resident vegetation cover, which included a mix of different weed species. Recently, the presence of orchards with a sown cover of different *Gramineae* species is also increasing in number. Both, resident or sown ground cover, are suggested by Integrated Citrus Pest Management Guidelines in Spain. Ground-cover vegetation attracts and harbors beneficial insects to the agrosystem, including pollinator insects (Nicholls and Altieri, 2013) and biological control agents such as parasitoids and predators that contribute to regulate pest populations (Aguilar-Fenollosa et al. 2011; Martínez-Ferrer et al. 2006; Silva et al. 2010). Beneficial insects may be exposed to neonicotinoids through contaminated plant-derived food sources such as nectar, pollen, guttation drops, or honeydew (Wäckers et al 2005, 2008; Heimpel and Jervis 2005; Urbaneja-Bernat et al 2020). The presence of wildflowers during all the year in the rows of the orchards favors the abundance and fitness of these entomophagous (Silva et al. 2010). *Cruciferae* species, for example, are plants that bloom in winter when food resources’ availability for pollinators and other insects is limited.

Integrated pest management (IPM) promotes the combination of all existing control methods and minimizes the use of pesticides. Thus, biological control plays a key role in IPM programs. However, pests that are not satisfactorily regulated by their natural enemies must be controlled by pesticides. Neonicotinoids, such as thiamethoxam and imidacloprid, are systemic (water-soluble insecticides that can move within plant vascular tissue) and persistent insecticides that are effective to control sap-sucking insects such as aphids, whiteflies, and mealybugs that are common pests in citrus crops (Cloyd and Bethke 2011; Grafton-Cardwell et al. 2008). These insecticides are applied in citrus crops as foliar or soil-treatments. When row crops are sprayed, a certain amount of the neonicotinoid insecticide reaches non-target areas, such as soil or the ground-cover vegetation, due to spray drift and foliar runoff by the action of air currents during the application. Garcerá et al. (2017) reported that after foliar application in citrus orchards with air-assisted axial-fan air blast sprayers, only around 46% of the applied spray was deposited on the target and 29% reaches the ground due to direct and indirect losses. Neonicotinoids that arrive to the ground could be highly persistent and can remain in the environment for years at low concentrations (Humaan-Guilleminot et al. 2019). Because neonocotinoids are highly water-soluble, they can move easily in the soil and arrive to non-target plants via uptake from roots (Goulson 2013). When neonicotinoid reach wildflowers, beneficial insects are exposed to them via plant-derived food sources (pollen, nectar, guttation, honeydew, vegetative tissues, etc.) that could lead to chronic exposures.

While there are studies on neonicotinoid contamination in wild plants growing near treated seed crops (Botías et al. 2015, 2016; David et al. 2016; Krupke et al. 2012; Long and Krupke 2016; Mogren and Lundgren 2016; Mörtl et al. 2019; Stewart et al. 2014), as far as the authors are aware, there is no available information on wildflower contamination due to the foliar application of neonicotinoids in tree crops under realistic field conditions. Therefore, the objective of this study was to evaluate and quantify thiamethoxam and imidacloprid in the whole wildflowers growing spontaneously in orchards after foliar application to citrus trees. The evaluation was carried out at different periods after neonicotinoid application. Research on the occurrence and concentrations of neonicotinoids in wildflowers in citrus orchards is essential in determining possible implications to biota, including entomophagous fauna, pollinators, and birds. It should be noted that this work was carried out before the prohibition by EU of the use of imidacloprid and thiamethoxam, except in permanent greenhouses, because they are a potential problem for honey bee viability (EFSA 2018a, 2018b). Nevertheless, many of the studies carried out to test neonicotinoids reported contradictory results regarding toxic effects on pollinators and beneficial insects. Moreover, the importance of this study relies on the fact that European agriculture only represents 4% of the global agricultural land (World Bank Group, 2021) and both insecticides are still frequently applied in citrus crops in different regions of the world. This work will add knowledge to elucidate the possible impact of neonicotinoids in field conditions that has not yet been fully established.

## Methods

### Orchards and application of neonicotinoids

The studies were carried out in eight commercial citrus orchards located in northeastern Spain (Table 1). The trees were grafted on citrange Carrizo. The orchards had 400–455 trees per ha, with a tree spacing of 5–5.5 × 4 m, with drip irrigation system. In each grove, an area corresponding to 360 trees was selected and divided into replicate plots that consisted of 30 (5 × 6) trees (600–660 m^2^). Four replicates of each treatment (untreated, thiamethoxam, and imidacloprid) were conducted. Replicate plots were randomly assigned in the selected area. The sprays were applied in a single application with Actara 25 WG from Syngenta (thiamethoxam 25% [WG]), 0.03% concentration or Confidor 20 LS from Bayer (imidacloprid 20% [WG]), and 0.075% concentration. Different volumes sprayed were applied on the orchards, depending on application parameters and the tree vegetation volume. Orchards 1, 2, 4, and 7 had a tree vegetation volume approximately of 10.800 m^3^ ha^−1^ and orchards 3, 5, 6, and 8 about 6.800 m^3^ ha^−1^. Spraying was performed as usual in commercial citrus orchards, with a 600L-turbo-sprayer Gaysa, at a pressure of 8–10 bars, speed of the tractor 2.2–2.7 km h^−1^ and 1140–1500 L ha^−1^. With these conditions, the total amount in average of thiamethoxam and imidacloprid was 0.085–0.113 and 0.171–0.227 kg ha^−1^, respectively (Table 1). In these citrus orchards, an IPM plan was followed and thiamethoxam or imidacloprid was not used at least 3 years before the application in these trial plots.

**Table 1:** Geolocation of orchards, date of application, date of sampling, treatment dose, and wildflower species collected in each orchard and sampling date

Orchard	Orchard 1	Orchard 2	Orchard 3	Orchard 4	Orchard 5	Orchard 6	Orchard 7	Orchard 8
Geolocation of orchards	40°30′35 N; 0°29′35 E	40°32′24 N; 0°27′08 E	40°30′54 N; 0°29′51 E	40°32′06 N; 0°26′48 E	40°52′01 N; 0°31′17 E	40°33′19 N; 0°25′39 E	40°30′16 N; 0°28′47 E	40°30′38 N; 0°29′36 E
Date of application	02/04/2015	13/05/2015	10/09/2015	26/04/2016	29/03/2017	29/03/2017	18/08/2016	20/05/2016
Date of sampling (dat)* Wildflower species	27/04/15 (25) *(a)* 02/06/16 (427) *C. arvensis*	22/05/15 (9) *(b)* 13/04/16 (336) *D. erucoides* *L. maritima* *S. tenerrimus*	27/4/16 (230) *D. erucoides* *S. tenerrimus*	20/05/16 (24) *D. erucoides* *L. maritima* *P. lanceolata* *S. tenerrimus* 06/6/16 (41) *C. arvensis* *D. erucoides* *P. lanceolata* *S. tenerrimus*	20/04/17 (22) *L. maritima* *S. tenerrimus*	19/04/17 (21) *D. erucoides* *S. tenerrimus*	07/09/16 (20) *L. maritima* 05/04/17 (230) *P. lanceolata* *S. tenerrimus*	13/06/17 (24) *C. arvensis* *L. maritima* *P. lanceolata* *S. tenerrimus* 21/04/17 (336) *D. erucoides* *L. maritima* *S. tenerrimus*
Imidacloprid a.i. ha^−1^ (Kg)	0.225	0.225	0.171	0.225	0.21	0.19	0.227	0.208
Thiamethoxam a.i. ha^−1^ (Kg)	0.113	0.113	0.085	0.113	0.105	0.095	0.1135	0.104

dat, days after treatment

*Veronica persica, Anagallis arvenis, S. tenerrimus, Capsella bursa pastoris, Malva sylvestris, Taraxacum officinale, Calendula arvensis, Erodium spp., L. marítima, C, arvensis* and *D. erucoides*

*S. tenerrimus, C. arvensis, L. maritima, Carduus nigrescens, D. erucoides* and *Melilotus lanceolata*

### Wildflower collection

Wildflowers, growing in citrus orchards previously treated with thiamethoxam or imidacloprid, were taken from the ground around the central trees of each replicate plot to avoid, as much as possible, the contamination by spray drift from the adjacent plots. Before flower sampling, a visual evaluation was carried out in each orchard to determine the wildflower species presence. Wildflower species were collected when the same species was present in at least 3 replicate plots by orchard. Up to four of the more representative species in each orchard were selected in every sampling. About 2 g of flowers, without their peduncles, were collected and stored in paper bags. Flowers were collected from the eight different citrus orchards at different periods after foliar citrus treatment (Table 1). The number of replicates and wildflower species was not the same in all plots, since they were not present naturally in all of them. Therefore, the sampled species of wildflowers depended on their availability in each orchard at the sampling periods. Weather conditions during 2015, 2016, and 2017 after neonicotinoid application were monitored (Table 2). In the first year of study (2015), in two occasions, a mixture of different wildflower species was sampled (Table 1). In the rest of the samplings, the wildflower species were *Plantago lanceolata, Convolvulus arvensis, Lobularia maritima, Diplotaxis erucoides*, and *Sonchus tenerrimus*.

**Table 2:** Rainfall (mm) recorded in the citrus orchards during the following 7 and 21 days after treatment and during the entire studied period (from the application to the sampling). Wind speed during the applications and during the following 24 h

Orchard	Application date	Sampling date	Dat	Rainfall (mm)			Wind speed (m/s) Mean (max)	
				7 dat	21 dat	Entire period	Application	24 h
1	02/04/15	27/04/15	25	0	4.4	20	0.86 (1.0)	0.5 (1.0)
		02/06/16	427	0	4.4	440		
2	13/05/15	22/05/15	9	6.6	6.6	6.6	1.9 (2.0)	0.9 (2.0)
		13/04/16	336	6.6	38.9	461		
3	10/09/15	27/04/16	230	16.4	54	246.5	2.0 (2.4)	1.3 (2.4)
4	26/04/16	20/05/16	24	5.3	35	38	1.8 (2)	1.2 (2)
		06/06/16	41	5.3	35	41.2		
5	29/03/17	20/04/17	22	0	0.2	0.2	0.5 (0.8)	0.9 (1.4)
6	29/03/17	19/04/17	21	0	0.2	0.2	0.5 (0.8)	0.9 (1.4)
7	18/08/16	07/09/16	20	0	1.7	1.7	2.4 (2.8)	1.3 (2.8)
		05/04/17	230	0	3.3	341.6		
8	20/05/16	13/06/16	24	2	4	4	2.0 (2.2)	1.3 (2.2)
		21/04/17	336	2	4	348.9		
		19/07/17	425	2	4	371.3		

Xarxa Agrometeorològica de Catalunya (Orchard 1: Aldover Station (40□52′47″ N; 0□29′57″ E). Orchards 2–8: Alcanar Station (40□33′13″ N; 0□30′55″ E)

### Analysis of neonicotinoid residues

Residue analysis of neonicotinoids was carried out using a QuEChERS (AOAC 2007) extraction method and quantification was performed by liquid chromatography with tandem mass spectrometry (LC–MS/MS). An Agilent system with a Model 1200 chromatograph and a Model 6410 triple quadrupole analyzer (Agilent Technologies, Palo Alto, CA, USA) was employed. LC analysis was performed with a F5 column of 100 × 3 mm i.d. and 2.6 μm, 100 Å particle size (Kinetex F5, Phenomenex, Torrance, CA, USA). The mobile phase A was 0.1% formic acid in water and mobile phase B was acetonitrile (ACN). The gradient used was as follows: 95% of A, decreased to 70% in 3 min, to 50% in 2 min, up to 2% in 3 min, and finally back to initial conditions in 4 min. The column was maintained at 25 °C, flow rate at 0.35 mL min^−1^, and the injection volume was 10 μL. The system used an electrospray ion source (ESI) operating in positive mode in the following conditions: drying gas temperature (300 °C), drying gas flow (10 Lmin^−1^), pressure of the nebulizer (40 psi), and capillary voltage (4000 V). Nitrogen gas was used in the nebulizer and in the collision cell. Identification and quantitation of neonicotinoid residues in samples of wildflowers were based on the detection of two selected reaction monitoring (SRMs transitions) one quantifier transition (SRM1) 292 > 211 and 256 > 175 and one qualifier transition (SRM2) 292 > 181 and 256 > 209 for thiamethoxam and imidacloprid, respectively.

Flowers (1 g) were weighed into a 30-mL polypropylene (PP) tube that contained two ceramic homogenizers, 4 mL of MilliQ water was added, and the PP tube was agitated in an automatic shaker for 5 min, in horizontal position. ACN (5 mL) was added and agitated for 2 min. A mixture of 2.5 g of anhydrous magnesium sulfate and sodium acetate (4: 1 w w^−1^) was added and the sample was vigorously shaken immediately and centrifuged for 5 min at 4500 rpm and 4 °C. An aliquot of the ACN extract (2 mL) was cleaned up with 200 mg of a mixture of PSA (primary secondary amine), C18, and graphitized carbon black (1:1:1, w w^−1^) shaking by vortex for 2 min and centrifuging for 5 min at 4500 rpm. An aliquot of the clean extract (1.0 mL) was evaporated to dryness in a vacuum evaporator (Genevac EZ-2, Ipswich, UK) and reconstituted in ACN:water (1:9) with the same volume. The extracts were filtered through a nylon filter 0.22 μm (Phenomenex, Torrance, CA, USA) before LC–MS/MS analysis. Recoveries at 1 ng g^−1^ (method quantification limit, MQL), 10 ng g^−1^ and 30 ng g^−1^, were from 90 to 112% and from 87 to 115% with a relative standard deviation (RSD) < 15% for thiamethoxam and imidacloprid, respectively. Wildflowers from control orchards were contaminated with neonicotinoids. Then, recoveries were carried out in wildflowers collected at 427 days after treatment (dat) where no neonicotinoids were found. Matrix match calibration in the range from 0 to 50 ng g^−1^ gave correlation coefficients > 0.99 for both analytes.

For quantitative residue analysis, as control wildflower samples were not free of neonicotinoids in many cases, a standard addition method was carried out by spiking the same volume of working standard solutions at different concentration levels into different aliquot extracts from each wildflower sample collected in each experimental plot (one aliquot was not spiked with the target analytes). In order to check if cross contamination occurred, all wildflower samples were analyzed for both neonicotinoids, thiamethoxam, and imidacloprid.

### Statistical analysis

All statistical analysis were conducted using Statgraphics Centurion XVII software. The non-parametric Mann–Whitney U test was used for the comparison of thiamethoxam and imidacloprid in overall samples and imidacloprid versus thiamethoxam in wildflower species. Kruskal–Wallis test was used to assess associations between wildflowers neonicotinoid levels and potential determinants of exposure such as different treated orchards, days after treatment, and wildflower species. For non-quantifiable detection or non-detection, concentration values were assuming of 0.15 ng g^−1^ (EPA, 2000).

## Results

Neonicotinoid analysis of wildflowers, based on pooled data, revealed contamination of both neonicotinoids throughout the study, with a higher proportion of samples contaminated with thiamethoxam than imidacloprid (72.8% versus 48.1%). Wildflowers had thiamethoxam concentration levels between < MQL (1 ng g^−1^) and 52.9 ng g^−1^, whereas imidacloprid ranged from < MQL to 98.6 ng g^−1^, when wildflowers were collected from citrus plots treated with thiamethoxam and imidacloprid, respectively (Table 3). It should be noted that only two of 103 samples had thiamethoxam concentration values > 38 ng g^−1^, and three samples from a total of 104 had imidacloprid concentration values > 38 ng g^−1^. Total imidacloprid concentration residues (median value of 1.0 and a mean ± SD of 4.8 ± 12.2) were significantly lower than thiamethoxam (median value of 3.4 and a mean ± SD of 6.8 ± 9.9) (Mann–Whitney test U = 4689.0; *P* = 0.001). Wildflower neonicotinoid concentrations in control plot samples ranged from < MQL to 4.7 ng g^−1^ for thiamethoxam and from < MQL to 3.4 ng g^−1^ for imidacloprid.

**Table 3:** Concentration levels and percentage of detection of thiamethoxam and imidacloprid in wildflowers collected from citrus orchards at different days after foliar treatment according to citrus orchard

		THIAMETHOXAM						IMIDACLOPRID					
	Days after treatment	9	22	41	230	336	427	9	22	41	230	336	427
Orchard 1	% detection > 1 ng g^−1^	-	100	-	-	-	0	-	100	-	-	-	0
	Range	-	3.6–8.0	-	-	-	< 1	-	4.2–8.5	-	-	-	< 1
	Median (ng g^−1^)	-	3.9	-	-	-	N/C	-	7.9	-	-	-	N/C
	Mean ± SD (ng g^−1^)	-	5.2 ± 2.5	-	-	-	N/C	-	6.8 ± 2.3	-	-	-	N/C
	Variation coefficient	-	47.6%	-	-	-	N/C	-	34.0%	-	-	-	N/C
	*N*	0	3	0	0	0		0	3	0	0	0	3
Orchard 2	% detection > 1 ng g^−1^	100	-	-	-	62.5	-	66.6	-	-	-	0	-
	Range	20.8–34.4	-	-	-	< 1–4.7	-	< 1–21.7	-	-	-	< 1	-
	Median (ng g^−1^)	22.8	-	-	-	1.5	-	10.7	-	-	-	N/C	-
	Mean ± SD (ng g^−1^)	26.0 ± 7.3	-	-	-	4.3 ± 5.9	-	11.0 ± 10.6	-	-	-	N/C	-
	Variation coefficient	28.2%	-	-	-	77.7%	-	97.0%	-	-	-	N/C	-
	*N*	3	0	0	0	7	0	3	0	0	0	8	0
Orchard 3	% detection > 1 ng g^−1^	-	-	-	100	-	-	-	-	-	50	-	-
	Range	-	-	-	2.6–6.2	-	-	-	-	-	< 1–5.0	-	-
	Median (ng g^−1^)	-	-	-	3.9	-	-	-	-	-	1.4	-	-
	Mean ± SD (ng g^−1^)	-	-	-	4.1 ± 1.5	-	-	-	-	-	3.3 ± 2.0	-	-
	Variation coefficient	-	-	-	36.9%	-	-	-	-	-	85.9%	-	-
	N	0	0	0	4	0	0	0	0	0	6	0	0
Orchard 4	% detection > 1 ng g^−1^	-	100	87.5	-	-	-	-	62.5	81.25	-	-	-
	Range	-	4.9–52.9	< 1–6.7	-	-	-	-	< 1–98.6	< 1–12.3	-	-	-
	Median (ng g^−1^)	-	13.8	3.2	-	-	-	-	3.8	2.8	-	-	-
	Mean ± SD (ng g^−1^)	-	20.0 ± 14.9	3.1 ± 1.7	-	-	-	-	15.6 ± 27.3	3.3 ± 2.9	-	-	-
	Variation coefficient	-	74.78%	54.5%	-	-	-	-	175.0%	87.0%	-	-	-
	N	0	16	16	0	0	0	0	16	16	0	0	0
□ Orchard 5	% detection > 1 ng g^−1^	-	57.2	-	-	-	-	-	42.8	-	-	-	-
	Range	-	< 1–19.1	-	-	-	-	-	< 1–12.6	-	-	-	-
	Median (ng g^−1^)	-	3.4	-	-	-	-	-	0.5	-	-	-	-
	Mean ± SD (ng g^−1^)	-	7.1 ± 7.7	-	-	-	-	-	2.8 ± 4.4	-	-	-	-
	Variation coefficient	-	108.3%	-	-	-	-	-	162.0%	-	-	-	-
	N	0	7	0	0	0	0	0	7	0	0	0	0
Orchard 6	% detection > 1 ng g^−1^	-	37.5	-	-	-	-	-	12.5	-	-	-	-
	Range	-	< 1–18.3	-	-	-	-	-	< 1–5.5	-	-	-	-
	Median (ng g^−1^)	-	1	-	-	-	-	-	0.5	-	-	-	-
	Mean ± SD (ng g^−1^)	-	3.7 ± 6.2	-	-	-	-	-	1.1 ± 1.7	-	-	-	-
	Variation coefficient	-	164.0%	-	-	-	-	-	157.0%	-	-	-	-
	N	0	8	0	0	0	0	0	8	0	0	0	0
Orchard 7	% detection > 1 ng g^−1^	-	100	-	0	-	-	-	66.7	-	0	-	-
	Range	-	1.9–20.3	-	< 1	-	-	-	< 1–8.2	-	< 1–1.0	-	-
	Median (ng g^−1^)	-	2.7	-		-	-	-	6.3	-	0.5	-	-
	Mean ± SD (ng g^−1^)	-	6.9 ± 8.9	-		-	-	-	5.0 ± 4.0	-	0.7 ± 0.3	-	-
	Variation coefficient	-	129.7%	-		-	-	-	80.0%	-	43.3%	-	-
	N	0	4	0	6	0	0	0	3	0	3	0	0
Orchard 8	% detection > 1 ng g^−1^	-	86.6	-	-	0	-	-	62.5	-	-	0	-
	Range	-	< 1–25.1	-	-	< 1	-	-	< 1–27.8	-	-	< 1	-
	Median (ng g^−1^)	-	3.5	-	-	N/C	-	-	2.6	-	-	N/C	-
	Mean ± SD (ng g^−1^)	-	5.8 ± 6.5	-	-	N/C	-	-	4.8 ± 7.3	-	-	N/C	-
	Variation coefficient	-	111.7%	-	-	N/C	-	-	148.9%	-	-	N/C	-
	N	0	15	0	0	11	0	0	16	0	0	12	0

*N/C*, not calculated

### Neonicotinoids in wildflowers depending on the different treated orchards

At 22 dat, wildflowers were sampled in 6 out of the 8 orchards (Table 3) and significant differences were found between the thiamethoxam residue levels in orchard 4 and the rest of orchards assessed (K–W = 19.4149; df = 5; *P* = 0.0016). For imidacloprid, although a higher average concentration level in orchard 4 was also observed, no significant differences between orchards were found (K–W = 9.52384; df = 5; *P* = 0.09).

When considering all orchards at 22 dat, except orchard 4, thiamethoxam average concentration levels ranged from 3.7 to 7.1 ng g^−1^ and imidacloprid from 1.1 to 6.8 ng g^−1^. In orchard 4, thiamethoxam and imidacloprid reached average concentration levels of 20.0 and 15.5 ng g^−1^, respectively.

### Neonicotinoids in wildflowers depending on the days after treatment

Thiamethoxam was present in wildflowers until 336 dat, and imidacloprid was detected until 230 dat. At 427 dat, neither neonicotinoids were detected above the MQL (Table 3).

**Fig. 1:**
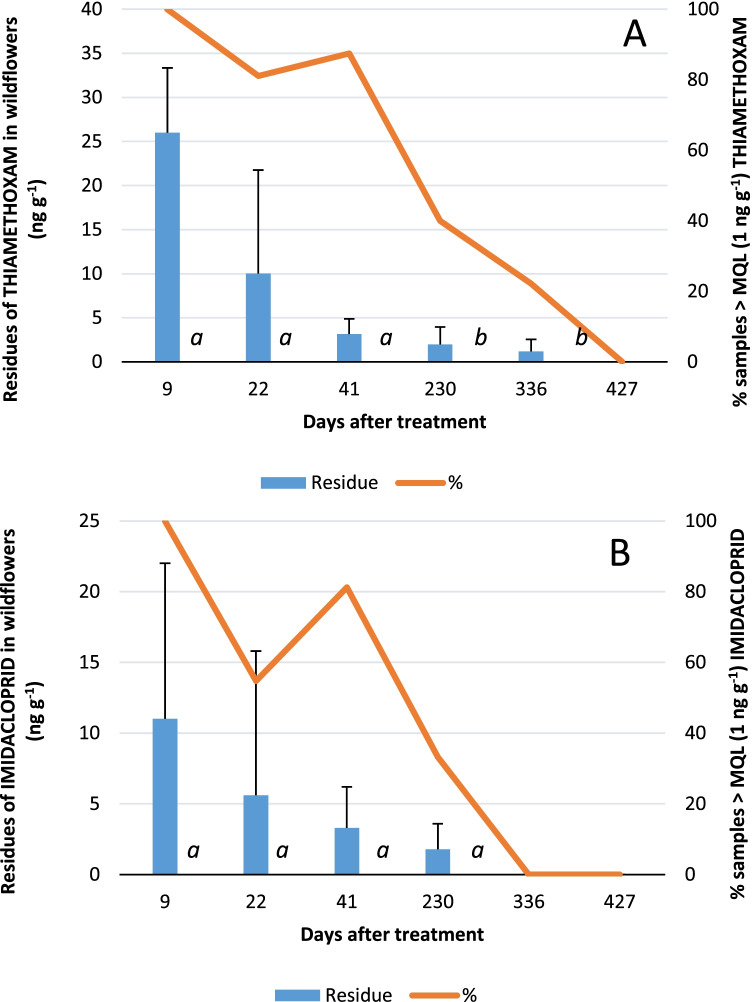
Average and standard deviation of **A** thiamethoxam and **B** imidacloprid concentration levels in wildflowers and percentage of samples with residues > MQL (1 ng g^−1^) depending on the days after treatment. Different letters mean significant differences (*P* < 0.05, Kruskal–Wallis tests)

The percentage of samples containing thiamethoxam residues remained similar from 9 to 41 dat, between 85 and 100% and no significant differences in concentration between 9, 22, and 41dat were observed. After that, the frequency of detection for thiamethoxam was almost halved from 41 to 230 dat, and from 230 to 336 dat, it was reduced again to about a half (Fig. 1A) being residues found at 230 and 336 dat significantly lower than those at 41dat (K–W = 29.1576; df = 4, 104; *P* < 0.0001).

The percentage of samples containing imidacloprid was similar at 9 and 41dat, between 80 and 100%, but at 22 dat, this percentage was lower than those. At 230 dat, a considerable reduction of the percentage of samples with imidacloprid was observed, and at 336 dat, no imidacloprid was detected in any wildflower sample (Fig. 1B). Although a clear decrease in the concentration of imidacloprid over time was observed (K–W = 18.3589; *df* = 4, 105; *P* = 0.0011), no significant differences were found among imidacloprid sampling periods.

The highest detection frequencies (100%), and highest thiamethoxam and imidacloprid concentrations, (26.0 ± 7.3 ng g^−1^ and 11.0 ± 10.6 ng g^−1^, respectively), occurred in wildflowers collected in days near neonicotinoid citrus application (9 dat).

### Neonicotinoids in wildflowers depending on species

From the total of wildflowers analyzed, the most abundant species was *S. tenerrimus* that represented the 33% of the total species sampled. *Convolvulus arvensis* was the less abundant species, 10.3% of the total.

Table S1 shows the concentration levels (ng g^−1^) at each time after neonicotinoid treatment for each wildflower species was analyzed and the frequency of detection.

At 22 dat, the five wildflower species considered in the study were sampled. At this dat, wildflower species accumulated different levels of imidacloprid (K–W = 17.41; df = 4; *P* = 0.004) and thiamethoxam (KW = 14.85; df = 4; *P* = 0.005). *Plantago lanceolata* accumulated the highest concentration of imidacloprid (31.4 ± 33.2 ng g^−1^) while *C. arvensis*, *D. erucoides*, and *L. maritima* are the lowest (1.7–2 ng g^−1^) (Table S1). Imidacloprid was quantified in 87.5% of *P. lanceolata*, 33.3% of *L. maritima*, 37.5% of *D. erucoides*, and 50% of *C. arvensis* samples. The highest residue levels of thiamethoxam at 22 dat were detected in *P. lanceolata* (19.7 ± 16.5) and *L. maritima* (14.9 ± 13.5 ng g^−1^) and the lowest, in *C. arvensis* (1.8 ± 1.9). Thiamethoxam was found in all *P. lanceolata* and *L. maritima* samples analyzed, whereas in *C. arvensis*, it was quantified in 66.7% of the analyzed samples.

To avoid the influence of the orchard in the concentration of neonicotinoids in the different species, wildflowers from the same orchard and dat were compared. In orchard 4, the residue levels among wildflower species at 22 dat and at 41 dat were compared, since at least four out of five different species could be sampled: *P. lanceolata*, *L. maritima (or C. arvensis)*, *S. tenerrimus*, and *D. erucoides*.

**Fig. 2:**
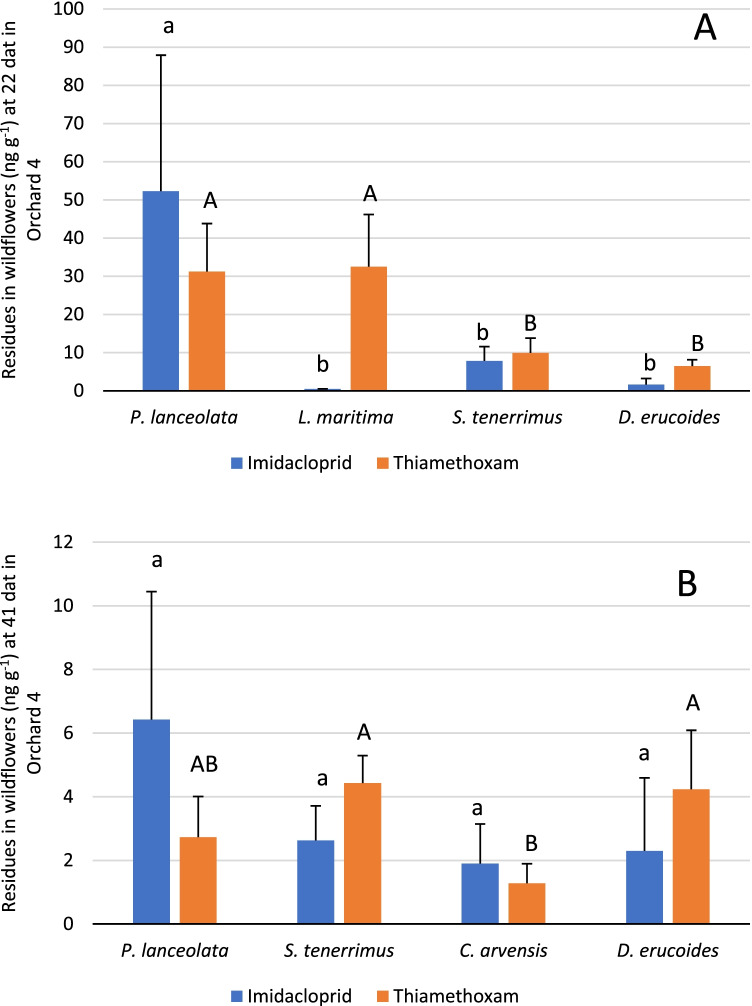
Average and standard deviation of thiamethoxam and imidacloprid concentration levels in four different wildflower species in orchard 4 at **A** 22 and **B** 41 days after treatment. Different letters indicate significant differences (*P* < 0.05, Kruskal–Wallis tests) between wildflower species with regard to imidacloprid (lowercase letters) and thiamethoxam (capital letters)

At 22 dat in orchard 4, significant differences in thiamethoxam and imidacloprid residue levels among wildflower species (thiamethoxam: K–W = 11.01; df = 3; *P* = 0.0092; imidacloprid: K–W = 13.42; df = 3; *P* = 0.0038) (Fig. 2A) were found. Imidacloprid residue levels in *P. lanceolata* were significantly higher than in the rest of the species that presented similar residues (*P* < 0.05) Thiamethoxam residue levels in *P. lanceolata* and *L. maritima* were similar and significantly higher than those found in *S. tenerrimus* and *D. erucoides* (*P* < 0.05) (Fig. 2A).

Thiamethoxam residue levels detected at 41 dat in orchard 4 were different among wildflower species (K–W = 8.98; df = 3; *P* = 0.03) but not those of imidacloprid ((K–W = 6.54; df = 3; *P* = 0.09). *Diplotaxis erucoides* and *S. tenerrimus* accumulated the highest thiamethoxam residue levels and *C. arvensis* accumulated the lowest (Fig. 2B).

Comparing the levels of imidacloprid and thiamethoxam in the same species collected at 22 dat in all orchards, imidacloprid was significantly lower than thiamethoxam in *L*. *maritima* (*U* = 1.5; *P* = 0.00002) and *D. erucoide*s (*U* = 7.0; *P* = 0.009), and no significant differences were observed between both neonicotinoids for *P. lanceolata* (*U* = 36.5; *P* = 0.674195), *S. tenerrimus* (*U* = 116.0; *P* = 0.89844), and *C. arvensis* (*U* = 6.0; *P* = 0.8544),

### Cross contamination

Contamination with imidacloprid was observed in wildflowers collected from plots treated with thiamethoxam and vice versa (Table S2).

**Fig. 3:**
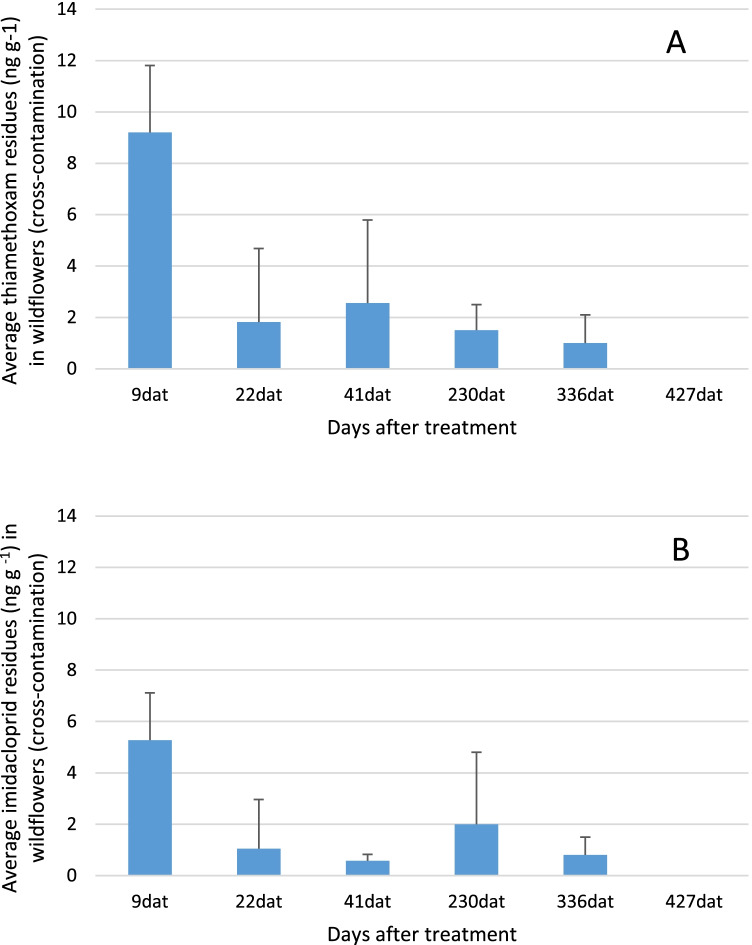
Average and standard deviation of **A** thiamethoxam and **B** imidacloprid concentration levels due to cross contamination in wildflowers from citrus orchards depending on the days after treatment

Overall, a higher concentration and frequency of detection for thiamethoxam were obtained in flowers collected from imidacloprid-treated plots than imidacloprid in flowers collected from thiamethoxam-treated plots (Fig. 3). Thiamethoxam was detected in 48.7% of wildflowers at 2.0 ± 2.8 ng g^−1^. Imidacloprid was detected in 15.5% of samples with 1.1 ± 1.8 ng g^−1^. The residue levels of thiamethoxam and imidacloprid detected due to cross contamination were 3.5- and fivefold lower than those found in plots directly sprayed with thiamethoxam and imidacloprid, respectively.

Values in Table S2 show a higher percentage of wildflowers contaminated by thiamethoxam than by imidacloprid at the same time and in the same orchard. The highest concentration and a 100% of neonicotinoid detection were obtained at 9 dat for both neonicotinoids. The mean concentration found at this time (9.2 ng g^−1^ of thiamethoxam and 5.3 ng g^−1^ of imidacloprid) represents around 35% of the thiamethoxam found in the plots treated with thiamethoxam, and around 48% of the imidacloprid in the plots treated with imidacloprid.

The detection frequency and concentration decreased considerably after 9 days of the application, with average concentration values between 1 and 2.5 ng g^−1^ for thiamethoxam and 0.6 and 2 ng g^−1^ for imidacloprid (Fig. 3). At 230 dat and 336 dat, there were still neonicotinoid residues in wildflowers collected in most of the sampled orchards.

## Discussion

The coefficient of variation for analysis of neonicotinoid residues indicates a high dispersion of concentration levels in wildflowers sampled, even when wildflowers were collected from the same orchard and at the same date (Table 3). Botías et al. (2015) also found highly variable concentrations of neonicotinoids in wildflowers collected from field margins, which were attributed to soil properties and environmental factors. The higher neonicotinoid concentrations found in orchard 4 at 22 dat, in relation to the rest of orchards, could be due to the rain that fell for 3 days immediately after the treatment that could have dragged part of pesticide from canopies to soil or/and to the groundcover vegetation (Table 2 and Table 3). In this way, the amount of rain fallen (15.6 mm) in the same day of application in orchard 3 could have increased the initial neonicotinoid amount in the soil by runoff from the trees with respect to orchard 7, where no rain events occurred until 22 dat (Table 2). Thus, neonicotinoids that fell to the soil could be bioavailable to plants longer than in orchard 7 at 230 dat (Table 2 and Table 3).

Similar difference between orchard 2 and 8 was also observed at 336 dat, probably due to rainfall in these orchards. Then, rainfall could be the main factor that explains the differences in concentration levels between orchards.

The highest concentration levels of both neonicotinoids during the first sampling (9 dat) followed of a decrease over time could indicate an initial contamination by direct deposition of neonicotinoids in wildflowers at the moment of application, as a consequence of the drift and foliar runoff due to spray application. Wildflowers sampled at 9 dat had a higher thiamethoxam residue level than imidacloprid.

In this study, at least near 336 days in the case of thiamethoxam and 230 days in the case of imidacloprid remained in the soil since they were detected in wildflowers and no more applications were made in that period. Neonicotinoids can remain in measurable concentrations for long periods in the soil, from 9 to 1250 days for imidacloprid and from 6 to 3001 days for thiamethoxam (Zhang et al. 2018).

Concentrations of neonicotinoid between 5 and 10 ppb are sufficient to protect plants against pests that feed on them (Goulson 2013). As shown by our results, the levels detected in wildflowers can often exceed this threshold. In the present study, 19.4% and 11.5% of samples exceeded 10 ppb of thiamethoxam and imidacloprid, respectively, and 32 and 21.1% exceeded 5 ppb, respectively. The results of this study indicate that these products could persist in soil and be taken up by wildflowers for such a long time (about 1 year). Parasitoids that visit these wildflowers could be feeding on nectar or pollen (Goulson 2013), on honeydew (Calvo-Agudo et al., 2019 and 2021), or on guttation droplets (Girolami et al., 2009) contaminated with imidacloprid, thiamethoxam, or both. Although the levels found may be low (< LC50), they could have side-effects due to a long-term exposure, as found in this work long after the neonicotinoid application. Estimated lethal concentration levels (LC_50_) for non-target insects are highly variable, as they can range from 31.29 ng/g to 2,630,000 for imidacloprid and from 16.91 to 1,440,000 for thiamethoxam (Botias et al., 2016).

In a previous study in these citrus orchards, it was found that nectar or pollen of citrus trees sprayed with imidacloprid and thiamethoxam, at the same doses that in this study, contained no neonicotinoid residues after 230 days (Martínez-Ferrer et al. 2019). The data provided in the present study on the neonicotinoid residue levels found in wildflowers at 230 and 336 days show that, in some cases, ground-cover vegetation may represent a more important route of pesticide chronic exposure for non-target arthropods than the crop itself.

When analyzing flowers in the margins of crop fields sown with neonicotinoid-treated seeds, the variability of neonicotinoid levels found is very wide. Mean concentration levels of 1.1 ± 6.0 ng g^−1^ of imidacloprid and 7.2 ± 31.9 ng g^−1^ of thiamethoxam were reported by Stewart et al. (2014), who analyzed neonicotinoids in wildflowers collected near seed-treated crops recently sown. Greatti et al. (2006) found imidacloprid residue levels from 22.4 to 123.7 ng g^−1^ in flowers growing near corn field with imidacloprid-treated seeds on the sowing day. Botías et al. (2016) reported neonicotinoid concentration levels between 0.02 and 106 ng g^−1^ in foliage from wild plants growing in the field margins of treated seed crops after 10 months, and Krupke et al. (2012) found neonicotinoid concentrations from 1.1 to 9.4 ng g^−1^ in *Taraxacum officinale* flowers collected a year after sowing seed-treated maize. In these studies, the levels of neonicotinoids found immediately after sowing the treated seeds or almost 1 year later do not appear to differ too much. However, in the present study, although a high variability in concentration levels was found, much higher levels (from < 1 to 98.6 ng g^−1^) were found when the flowers were sampled closer to foliar application (at 22 dat, Table 3) than 1 year (336 dat) since application (< 1–4.7 ng g^−1^).

At 22 dat, when all the wildflower species included in this work could be compared, different neonicotinoid levels between them were found, indicating that the accumulation of each neonicotinoid depends on the species. Differences in neonicotinoid concentrations in pollen and nectar of citrus flowers from different varieties were also found after neonicotinoid application (Martínez-Ferrer et al. 2019). Li et al. (2018) reported that the amount of imidacloprid taken up by vegetables differed with the variety of vegetable and its growth stage, and it is related to the evapotranspiration and availability of imidacloprid in roots. In this work, different wildflower species growing in the same orchard with the same environmental conditions contained different concentrations of neonicotinoids. Therefore, differences among plant species could be related to the physiological mechanisms that regulate absorption, translocation, and dissipation in each wildflower species. Although the differences in the uptake and translocation of various neonicotinoids in plants are still not clear (Li et al., 2018), differences among plant species in longevity, relative growth rate, or root morphology might affect the uptake capacities and the metabolic pathways of neonicotinoids (Botías et al., 2016).

The manipulation of the composition of groundcover within orchards is a habitat management technique that might enhance biological control of orchard arthropod pests (Prokopy 1994). The differences on the neonicotinoid residue levels among wildflower species found in the present study suggest that this factor should not be overlooked when selecting the wildflower species that compose the ground-cover vegetation. Long and Krupke (2016) reported that honeybee foragers collected a greater amount of *Brassicacceae* (up to 12.6%) and *Plantagineaceae* (up to 10.99%) pollen in relation to other plant families. Most of the flowers analyzed in the present study belong to these families (*Diplotaxis, Lobularia*, and *Plantago)*; therefore, they can constitute a risk for arthropods, especially in periods close to foliar application of neonicotinoids where the residues in these flowers were higher.

Spray drift and environmental conditions, during neonicotinoid application to citrus, seem responsible of an initial relatively high cross contamination that decreases with time. After this time (9 dat), cross contamination was probably a consequence of runoff in soil from the treated adjacent plots and the persistence of these pesticides in soil. This cross contamination, probably due to these same factors, was also observed in control samples which could not be used to correct the values obtained in wildflowers collected from treated orchards. Currently, citrus orchards are managed very intensively, with mature trees forming a near continuous-row canopy, which can have a crosswise mid-width of up to 3.0 m. In the present study, the minimum distance between plots in the assessed orchards was 16.5 m.

It should be noted that, although the dose of imidacloprid applied was almost two-fold higher than for thiamethoxam (Table 1), thiamethoxam concentrations and frequency of detection in wildflowers were higher than those for imidacloprid. Thiamethoxam levels were also higher than those of imidacloprid, in relation to the applied dose, in the pollen of orange blossom flowers assayed in these citrus orchards and published in a previous work (Martinez-Ferrer et al. 2019). Hladik et al. (2014) in streams from corn and soybean crops also found that the frequency of occurrence of imidacloprid and thiamethoxam was reverse to the amount applied. These results suggest that thiamethoxam has greater tendency to be taken up by plants than imidacloprid, probably due to its higher water solubility (4100 mg L^−1^ versus 610 mg L^−1^) and their lower organic carbon–water partition coefficient, − 0.13 for thiamethoxam and 0.57 for imidacloprid (Lewis et al. 2016). In addition, when foliar application was carried out, a great exposure of neonicotinoids to sunlight takes place. Imidacloprid photolysis degradation is higher than that of thiamethoxam (aqueous photolysis DT50 at pH 7: 0.2 days versus 2.7 days (Lewis et al., 2016), being able to make imidacloprid less available to plants. Photolysis could be one of the factors that contributed to this fact. In addition to the aforementioned main factor, the rainfall, other factors that affect the mobility and dissipation of neonicotinoids in soil are wind erosion and land slope (Limay-Rios et al. 2016; Niu et al. 2020), biological degradation dependent of microorganisms on each soil orchard (Hilton et al. 2016; Liu et al. 2001) soil type, cracks and macropores, and temperature (Chrétien et al. 2017; Mörtl et al. 2016; Radolinski et al. 2018; Yadav and Watanabe 2018). Therefore, all these factors in greater or lesser intensity could have affected the results obtained.

## Conclusions

Citrus entomofauna and pollinators feed on wildflowers all year long. Furthermore, in combination to the wildflowers, they feed on citrus flowers, but this only happens during the brief flowering period of the citrus crops (about 1 month). These plant-derived food sources could be contaminated by neonicotinoids and may affect their health. The wildflowers associated with the citrus orchards studied in this work were contaminated by the foliar application of thiamethoxam and imidacloprid and this contamination was detected until at least 336 and 230 dat, respectively, but not after 427 dat. Wildflower contamination was produced directly, due to spray drift and foliar runoff of these pesticides, and indirectly, by uptake from soil after its application to the tree canopy being the rainfall an important factor that contributes to that fact. In addition, these neonicotinoids may reach other areas where wildflowers are present due to their persistence and mobility in soil.

In the present study, different residue levels were found depending on the days after treatments and the wildflower species. Thiamethoxam was the insecticide most frequently detected with the highest residue levels in comparison to imidacloprid. In citrus IPM programs, it is highly recommended to maintain a cover crop in the alleyways of the orchards to enhance biodiversity and improve biological control. However, in this study, it is demonstrated that maintaining a cover crop in citrus orchards, when they are treated with neonicotinoid, may lead to adverse effects on non-target arthropods due to a prolonged exposure because these products were detected at least 230 dat for imidacloprid and 336 dat for thiamethoxam. The consequences of this long exposure at field level should be studied in the entomofauna and the different response found according to wildflower species should be considered when selecting the ground-cover species composition.

Cross contamination over time was revealed in plots 16.5 m away, indicating a transport from adjacent treated plots, probably by runoff and soil dust that together the persistence of these pesticides in soil make them available to be taken up by plants.

## Supplementary Information

Below is the link to the electronic supplementary material.

Supplementary file1 (DOCX 29 KB)

## Data Availability

Not applicable.
