# Supplementary material for: Neonicotinoid contamination in wildflowers collected from citrus orchards in a northwestern Mediterranean Region (Spain) after tree foliar treatments
Source: Environ Sci Pollut Res Int. 2022 Mar 14;29(35):53482–95. doi: 10.1007/s11356-022-19331-7 (PMC9343284; doi:10.1007/s11356-022-19331-7)
Supplement: Supplementary file 1 — Supplementary file1 (DOCX 29 KB) [file 11356_2022_19331_MOESM1_ESM.docx]

**Table S1.** Concentration levels and percentage of detection of thiamethoxam and imidacloprid in wildflowers collected from citrus orchards at different days after foliar treatment (dat) according to wildflowers species.

|  |  | THIAMETHOXAM | | | | | IMIDACLOPRID | | | | |
| --- | --- | --- | --- | --- | --- | --- | --- | --- | --- | --- | --- |
|  | Days after treatment | 22 | 41 | 230 | 336 | 427 | 22 | 41 | 230 | 336 | 427 |
| *C. arvensis* | % detection > 1ng g^-1^ | 33.3 | 75 | - | - | 0 | 50 | 75 | - | - | 0 |
|  | Range | <1-4.0 | <1-1.9 | - | - | <1 | <1-4.2 | <1-3.5 | - | - | <1 |
|  | median (ng g^-1^) | 1 | 1.35 | - | - | N/C | 1.6 | 1.8 | - | - | N/C |
|  | mean±SD (ng g^-1^) | 1.8±1.9 | 1.3±0.6 | - | - | <1 | 2.0±1.8 | 1.9±1.2 | - | - | <1 |
|  | Variation coefficient | 103.3 | 48.1 | - | - | N/C | 91.4 | 65.3 | - | - | N/C |
|  | N | 3 | 4 | - | - | 3 | 4 | 4 | - | - | 3 |
|  |  |  |  |  |  |  |  |  |  |  |  |
| *D. erucoides* | % detection > 1ng g^-1^ | 87.5 | 100 | - | 20 | - | 37.5 | 50 | 66.7 | 0 | - |
|  | Range | 1-18.3 | 2.6-6.7 | 3.5-3.5 | <1-1.5 | - | <1-5.5 | <1-5.3 | <1-5 | <1 | - |
|  | median (ng g^-1^) | 5.9 | 3.8 | - | 0.5 | - | <1 | 1.7 | 4.8 | N/C | - |
|  | mean±SD (ng g^-1^) | 6.6±5.4 | 4.2±1.9 | 3.5±0 | 0.8±0.5 | - | 1.7±1.9 | 2.3±2.3 | 3.4±2.5 | N/C | - |
|  | Variation coefficient | 81.0 | 44.1 | - | 55.9 | - | 112.0 | 99.9 | 74.1 | N/C | - |
|  | N | 8 | 4 | 1 | 5 | - | 8 | 4 | 3 | 6 | - |
|  |  |  |  |  |  |  |  |  |  |  |  |
| *L. maritima* | % detection > 1ng g^-1^ | 100 | - | - | 33.3 | - | 33.3 | - | - | 0 | - |
|  | Range | 1.9-52.9 | - | - | <1-4.7 | - | <1-8.2 | - | - | <1 | - |
|  | median (ng g^-1^) | 13 | - | - | 0.5 | - | 0.5 | - | - | N/C | - |
|  | mean±SD (ng g^-1^) | 14.9±13.5 | - | - | 1.6±1.8 | - | 1.7±2.4 | - | - | N/C | - |
|  | Variation coefficient | 90.6 | - | - | 113.4 | - | 141.5 | - | - | N/C | - |
|  | N | 16 | - | - | 6 | - | 15 | - | - | 7 | - |
|  |  |  |  |  |  |  |  |  |  |  |  |
| *P. lanceolata* | % detection > 1ng g^-1^ | 100 | 75 | 0 | - | - | 87.5 | 100 | - | - | - |
|  | Range | 1.7-40.5 | 1.0-3.7 | <1 | - | - | <1-98.6 | 3.5-12.3 | - | - | - |
|  | median (ng g^-1^) | 18.85 | 3.1 | N/C | - | - | 20.2 | 5.0 | - | - | - |
|  | mean±SD (ng g^-1^) | 19.7±16.5 | 2.7±1.3 | <1 | - | - | 31.4±33.2 | 6.4±4.0 | - | - | - |
|  | Variation coefficient | 83.9 | 47.0 | N/C | - | - | 105.4 | 62.6 | - | - | - |
|  | N | 8 | 4 | 3 | - | - | 8 | 4 | - | - | - |
|  |  |  |  |  |  |  |  |  |  |  |  |
| *S. tenerrimus* | % detection > 1ng g^-1^ | 53.3 | 100 | 50 | 14.3 | - | 60 | 100 | 16.7 | 0 | - |
|  | Range | <1-15.0 | 3.4-5.5 | <1-6.2 | <1-4.6 | - | <1-13.7 | 1.5-4.1 | <1-1.8 | <1 | - |
|  | median (ng g^-1^) | 2.1 | 4.4 | 1.5 | 0.5 | - | 3.6 | 2.45 | 1.00 | N/C | - |
|  | mean±SD (ng g^-1^) | 4.1±4.5 | 4.4±0.9 | 2.4±2.4 | 1.2±1.5 | - | 4.8±4.9 | 2.6±1.1 | 1.0±0.5 | N/C | - |
|  | Variation coefficient | 110.3 | 19.4 | 98.8 | 141.5 | - | 102.4 | 41.4 | 49.3 | N/C | - |
|  | N | 15 | 4 | 6 | 7 | - | 15 | 4 | 6 | 7 | - |

N/C: not calculated

**Table S2.** Cross contamination: Concentration levels and percentage of detection of thiamethoxam in wildflowers collected from citrus orchards at different days after foliar treatment with imidacloprid and vice versa.

|  |  |  | THIAMETHOXAM | | | | | | IMIDACLOPRID | | | | | |
| --- | --- | --- | --- | --- | --- | --- | --- | --- | --- | --- | --- | --- | --- | --- |
|  |  | | Imidacloprid treatment | | | | | | Thiamethoxam treatment | | | | | |
|  | Days after treatment | | 9 | 22 | 41 | 230 | 336 | 427 | 9 | 22 | 41 | 230 | 336 | 427 |
| Orchard 1 | % detection > 1ng g^-1^ | | - | 33.3 | - | - | - | 0 | - | 100 | - | - | - | 0 |
|  | range | | - | <1-1.1 | - | - | - | <1 | - | 1.9-9.6 | - | - | - | <1 |
|  | median (ng g^-1^) | | - | 1 | - | - | - | N/C | - | 8.7 | - | - | - | N/C |
|  | mean±SD (ng g^-1^) | | - | 0.9±0.3 | - | - | - | N/C | - | 6.7±4.2 | - | - | - | N/C |
|  | Variation coefficient | | - | 37.1% | - | - | - | N/C | - | 63% | - | - | - | N/C |
|  | N | | 0 | 3 | 0 | 0 | 0 | 3 | 0 | 3 | 0 | 0 | 0 | 3 |
|  |  | |  |  |  |  |  |  |  |  |  |  |  |  |
| Orchard 2 | % detection > 1ng g^-1^ | | 100 | - | - | - | 50 | - | 100 | - | - | - | 28.5 | - |
|  | range | | 6.2-10.9 | - | - | - | <1-3.9 | - | 4.1-7.4 | - | - | - | <1-1.9 | - |
|  | median (ng g^-1^) | | 10.5 | - | - | - | 0.5 | - | 4.3 | - | - | - | 0.5 | - |
|  | mean±SD (ng g^-1^) | | 9.2±2.6 | - | - | - | 1.7±154 | - | 5.3±1.8 | - | - | - | 0.8±0.6 | - |
|  | Variation coefficient | | 28.3% | - | - | - | 85.9% | - | 35.0% | - | - | - | 68.8% | - |
|  | N | | 3 | 0 | 0 | 0 | 8 | 0 | 3 | 0 | 0 | 0 | 7 | 0 |
|  |  | |  |  |  |  |  |  |  |  |  |  |  |  |
| Orchard 3 | % detection > 1ng g^-1^ | | - | - | - | 100 | - | - | - | - | - | 50 | - | - |
|  | range | | - | - | - | 1.2-3.3 | - | - | - | - | - | <1-1.9 | - | - |
|  | median (ng g^-1^) | | - | - | - | 2.1 | - | - | - | - | - | 0.8 | - | - |
|  | mean±SD (ng g^-1^) | | - | - | - | 2.1±0.9 | - | - | - | - | - | 1.0±0.7 | - | - |
|  | Variation coefficient | | - | - | - | 42.4% | - | - | - | - | - | 67.7% | - | - |
|  | N | | 0 | 0 | 0 | 6 | 0 | 0 | 0 | 0 | 0 | 4 | 0 | 0 |
|  |  | |  |  |  |  |  |  |  |  |  |  |  |  |
| Orchard 4 | % detection > 1ng g^-1^ | | - | 50 | 75 | - | - | - | - | 25 | 6.2 | - | - | - |
|  | range | | - | <1-3.6 | <1-13.9 | - | - | - | - | <1-7.4 | <1-1.5 | - | - | - |
|  | median (ng g^-1^) | | - | 0.8 | 1.8 | - | - | - | - | 0.5 | 0.5 | - | - | - |
|  | mean±SD (ng g^-1^) | | - | 1.2±0.9 | 2.5±3.2 | - | - | - | - | 1.2±1.7 | 0.6±0.2 | - | - | - |
|  | Variation coefficient | | - | 76.8% | 126.8% | - | - | - | - | 148.0% | 44.0% | - | - | - |
|  | N | | 0 | 16 | 16 | 0 | 0 | 0 | 0 | 16 | 16 | 0 | 0 | 0 |
|  |  | |  |  |  |  |  |  |  |  |  |  |  |  |
| Orchard 5 | % detection > 1ng g^-1^ | | - | 0 | - | - | - | - | - | 0 | - | - | - | - |
|  | range | | - | <1 | - | - | - | - | - | <1 | - | - | - | - |
|  | median (ng g^-1^) | | - | N/C | - | - | - | - | - | N/C | - | - | - | - |
|  | mean±SD (ng g^-1^) | | - | N/C | - | - | - | - | - | N/C | - | - | - | - |
|  | Variation coeficient | | - | N/C | - | - | - | - | - | N/C | - | - | - | - |
|  | N | | 0 | 7 | 0 | 0 | 0 | 0 | 0 | 7 | 0 | 0 | 0 | 0 |
|  |  | |  |  |  |  |  |  |  |  |  |  |  |  |
| Orchard 6 | % detection > 1ng g^-1^ | | - | 0 | - | - | - | - | - | 0 | - | - | - | - |
|  | range | | - | <1 | - | - | - | - | - | <1 | - | - | - | - |
|  | median (ng g^-1^) | | - | N/C | - | - | - | - | - | N/C | - | - | - | - |
|  | mean±SD (ng g^-1^) | | - | N/C | - | - | - | - | - | N/C | - | - | - | - |
|  | Variation coefficient | | - | N/C | - | - | - | - | - | N/C | - | - | - | - |
|  | N | | 0 | 8 | 0 | 0 | 0 | 0 | 0 | 8 | 0 | 0 | 0 | 0 |
|  |  | |  |  |  |  |  |  |  |  |  |  |  |  |
| Orchard 7 | % detection > 1ng g^-1^ | | - | 0 | - | 33.3 | - | - | - | 0 | - | 50 | - | - |
|  | range | | - | <1 | - | <1-1.2 | - | - | - | <1 | - | <1-8.2 | - | - |
|  | median (ng g^-1^) | | - | N/C | - | 0.5 | - | - | - | N/C | - | 0.5 | - | - |
|  | mean±SD (ng g^-1^) | | - | N/C | - | 0.7±0.4 | - | - | - | N/C | - | 3.2±3.7 | - | - |
|  | Variation coefficient | | - | N/C | - | 55.1 | - | - | - | N/C | - | 117.3% | - | - |
|  | N | | 0 | 3 | 0 | 3 | 0 | 0 | 0 | 4 | 0 | 6 | 0 | 0 |
|  |  | |  |  |  |  |  |  |  |  |  |  |  |  |
| Orchard 8 | % detection > 1ng g^-1^ | | - | 75 | - | - | 0 | - | - | 0 | - | - | 9.1 | - |
|  | range | | - | <1-14.6 | - | - | <1 | - | - | <1 | - | - | <1-3.0 | - |
|  | median (ng g^-1^) | | - | 2.8 | - | - | N/C | - | - | N/C | - | - | 0.5 | - |
|  | mean±SD (ng g^-1^) | | - | 4.1±4.4 | - | - | N/C | - | - | N/C | - | - | 0.7±0.7 | - |
|  | Variation coefficient | | - | 108.6% | - | - | N/C | - | - | N/C | - | - | 104.0% | - |
|  | N | | 0 | 16 | 0 | 0 | 12 | 0 | 0 | 15 | 0 | 0 | 11 | 0 |

N/C: not calculated
